# Editorial: Development of Humanized Mouse Models for Infectious Diseases and Cancer

**DOI:** 10.3389/fimmu.2019.03051

**Published:** 2020-01-10

**Authors:** Moriya Tsuji, Ramesh Akkina

**Affiliations:** ^1^Aaron Diamond AIDS Research Center, Affiliate of the Rockefeller University, New York, NY, United States; ^2^Department of Microbiology, Immunology and Pathology, Colorado State University, Fort Collins, CO, United States

**Keywords:** humanized mice in infectious disease research, humanized mice in cancer research, new humanized mouse models, humanized mice for HIV, malaria, vaccines and therapeutics, improved humanized mouse models

Many knowledge gaps exist in translating research results from conventional animal models such as mice to the human, especially in the clinical context. *In vivo* systems incorporating human cells and tissues in a physiological setting will help bridge this gap. In this regard, humanized mice with engrafted human cells provide suitable tools to study human specific pathogens and cancer. With a transplanted human immune system, they also offer a dynamic setting for immune responses. Central to the preparation of new generation humanized mice is the availability of various strains of immunodeficient mice. Many new advances in this arena include derivation of mouse strains transgenic for human cytokines and HLA alleles, allowing improved human cell engraftment and immune responses. Transplantation of tissues such as human liver together with an autologous immune system paved the way for new studies not previously feasible. Human specific pathogens such as HIV, hepatitis viruses, and malaria parasites are being intensely studied in these systems and important data on pathogen life cycles, viral latency, and human specific immune responses are gathered. In the cancer field, patient derived xenograft models are facilitating testing of various chemo- and immunotherapies. Recent applications of these models expanded immensely to address host-parasite interactions involving more diverse agents and in studying viral-bacterial co-infections as well. Studies on novel gene, cellular and antibody therapies have also greatly expanded by the use of these mice.

The current Research Topic incorporates a number of original research papers and review articles addressing a wide range of topics that include new model development, viruses, bacteria, parasites, cancer, and vaccine studies demonstrating the ever increasing versatility of humanized mice in biomedical research.

Two humanized mouse models are widely used in HIV research, the simpler and less expensive hu-HSC mouse model and the BLT hu-mouse model requiring surgery to transplant human thymic tissue and hematopoietic stem cells (HSC). Cheng et al. compared immune reconstitution and HIV-1 infection between NRG-huHSC and NRG-Hu Thy/HSC models. Interestingly, both models were found to support comparable levels of virus replication, immunopathology, and therapeutic responses to ART and immunotherapy approaches suggesting that hu-HSC mice can be effectively used in many HIV experimental settings with reduced cost and labor. Soper et al. reviewed the impact of type I IFN (IFN-I) in HIV-1 infection *in vivo* utilizing hu-HSC mouse models. They found that the effects of IFN-I in the *in vivo* context were much more complicated than previously predicted from *in vitro* studies, thus underscoring the advantage of using humanized mouse models in assessing the nuances of IFN-I effects for/against viral infections.

A major goal in the HIV/AIDS field is to achieve full viral eradication and a complete cure. However, this has been elusive due to the presence of minute levels of latently infected cells even in fully virus suppressed patients on long-term therapy. Ultra-sensitive assays are needed to verify when full HIV remission is achieved. Schmitt and Akkina reviewed the current status of HIV latency detection assays and discussed the higher sensitivity achieved utilizing humanized mouse-based viral outgrowth assays (hmVOA) vs. *in vitro* VOAs.

In the context of HIV-1 viral persistence, the central nervous system (CNS) has come into the limelight as a unique, immunologically privileged compartment supporting infection and consequent immune-mediated damage. Evering and Tsuji reviewed the current work on HIV-1 in the CNS using human immune system (HIS) mouse models with a focus on cells of myeloid lineage playing a major role. They predict that the new HIS mouse models in the current pipeline will further facilitate novel diagnostic, therapeutic, and viral eradication strategies in the CNS.

Lentiviral gene transduction of human hematopoietic cells including HSC opened up many avenues of gene and cellular therapies. Humanized mice played an ever increasing role in modeling these new strategies and providing important pre-clinical data. Carrillo et al. reviewed recent developments in CAR-T cell-based immunotherapies and their combination with antibody targeting of immune checkpoint inhibitors such as PD-1. Stem-cell based approaches using TCRs against HIV and cancer were discussed. Hyperimmune activation during HIV-1 infection appears to be driven by chronic IFN-I induction. Hu-mouse studies determined that blocking the IFN-I signaling by antibodies decreased immune activation and resulted in reversal of T cell exhaustion.

Kim et al. described the use of humanized DRAG mice (HLA class II DR4 transgenic) for HIV-1 transmission via intravaginal route. Superior human cell engraftment in mucosal sites was noted. Viral spread from the point of entry were studied in detail with the results supporting the utility of this improved model to study viral pathogenesis, tissue distribution, viral persistence and establishment of latent viral reservoirs. Volk et al. described a multidimensional analysis approach integrating human T cell signatures in lymphatic tissues with the sex of humanized mice as a predictor of responses after dendritic cell-based immunization. This new modality of multidimensional analysis can be potentially used as a framework for assessing predictive signatures of immune responses.

Viral hemorrhagic fevers (VHF) such as Ebola, Dengue, and Crimean-Congo hemorrhagic fever with high fatality rates constitute important public health concerns. While the natural hosts for these viruses in the wild are asymptomatic, humans are severely affected, incriminating a role for the human immune system in mediating severe pathology. Schönrich and Raftery reviewed the impact of humanized mice in VHF vaccines and therapeutics research, also emphasizing their role as surrogate models for the discovery of newly emerging zoonotic agents.

Hu-mice provide excellent models to study tumorigenesis and immune responses. Among the virus-related human cancers, EBV and KSHV account for 10% of morbidity. Their epidemiology varies drastically in different geographic regions. The review by Münz detailed the tumorigenesis by these viruses, interesting aspects of how KSHV infection is sustained longer during EBV coinfection in hu-mice, how the adaptive and innate immune responses play out and how this knowledge can be used to develop effective vaccines in the future. With regard to modeling anti-tumor responses *in vivo* in humanized mice, Zumwalde and Gumperz reviewed the use of humanized mice engrafted with human umbilical cord blood-derived HSC. They also discussed how T cells get suppressed during EBV tumorigenesis and how immunotherapy strategies can counteract this.

The tumor microenvironment contains unique immune cells, termed myeloid-derived suppressor cells (MDSC) and tumor-associated macrophages (TAM) that suppress host anti-tumor immunity and promote tumor angiogenesis and metastasis. Hanazawa et al. described the generation of a functional human TAM population in their novel humanized IL-6 transgenic mouse strain, NOG-hIL-6 Tg. Development of novel cancer immune therapies targeting immunoregulatory/immunosuppressive myeloid cells is now possible using this model. The same research group led by Takahashi et al. reported the derivation of a new transgenic mouse strain, NOG-pRORγt-γc, in which the γc gene was expressed in a lymph-tissue inducer (LTi) lineage by the endogenous promoter of RORγt. Lymph node development was greatly improved, a major deficiency with previous HIS mouse models. Increased numbers of IL-21–producing CD4+ T cells were seen in LNs and there was enhanced antigen specific IgG response thus providing a vastly improved HIS mouse model.

Two reports focused on bacterial studies. *Staphylococcus aureus* is an important human pathogen responsible for many disease conditions including fatal pneumonia and septicemia. While conventional mice have been useful to study these conditions to an extent, it has become clear that some virulence factors/toxins display higher specificity to the human cells/factors leading to more severe disease. Parker's review highlights the value of humanized mice in dissecting the role of *S. aureus* virulence factors in a human surrogate setting and in vaccine testing.

Over one million people worldwide are affected annually by Scrub typhus, a disease caused by an intracellular bacterium *Orientia tsutsugamushi*. Although standard mouse models provided a basic understanding, data is sparse on human immunopathogenesis and immune responses. Jiang et al. described the successful use of a humanized DRAGA (HLA-A2 and HLA-DR4-transgenic) mouse model capable of efficient human cellular and antibody responses. Footpad infection with *O. tsutsugamushi* resulted in disseminated lesions in various organs and invoked human immune responses including T cell activation, specific antibody and cytokine secretion mimicking human disease and responses. Vaccination with killed whole cell *O. tsutsugamushi* gave rise to both humoral and cellular responses thus providing a human relevant model for future vaccines and therapeutics testing.

Malaria continues to inflict high morbidity and mortality numbering in millions in many parts of the world. Transmitted by mosquitoes, the parasite has a complex life cycle with many stages of development. Minkah et al. reviewed the current status of malaria animal models and point to the need to develop humanized mouse models that can support both the hepatic and blood stages of infection to study pathogenesis and enable therapeutic testing. With these criteria as a background, the report by Foquet et al. described establishment of a FRGN huHep/hRBC humanized mouse model. This animal model enabled human malaria parasites to successfully undergo the liver stages and culminate with the blood stages of infection *in vivo*. Imaging techniques used to test the efficacy of an inhibitory monoclonal antibody demonstrated the utility of the model in evaluating interventions that target one or both phases of the parasite life cycle.

In summary, this Research Topic highlights the recent advancements in biomedical research using different models of humanized mice. As can be seen, these models have been substantially improved over the past decade increasing their breadth in utility not only in studying the infection process of the pathogens but also allowed evaluation of host immune responses thus laying a foundation to build upon for future vaccine and therapeutic testing.

We thank all the authors of the manuscripts for their contributions to the humanized mouse field and reviewers for their constructive comments and input.

## Author Contributions

All authors listed have made a substantial, direct and intellectual contribution to the work, and approved it for publication.

### Conflict of Interest

The authors declare that the research was conducted in the absence of any commercial or financial relationships that could be construed as a potential conflict of interest.

